# A135 DO VISCOUS SWALLOWS IMPROVE THE DIAGNOSTIC YIELD OF HIGH-RESOLUTION MANOMETRY?

**DOI:** 10.1093/jcag/gwab049.134

**Published:** 2022-02-21

**Authors:** M Ricci, D Rodrigues, D E Reed

**Affiliations:** Queen’s University, Kingston, ON, Canada

## Abstract

**Background:**

Esophageal motility disorders are diagnosed using high-resolution esophageal manometry (HR-EMS) using the Chicago Classification (CC V4.0) which is based on a series of 10 normal saline swallows (LS). Viscous swallows (VS; thickened liquid/applesauce) are often performed during esophageal manometry; however, they were not included within the framework of CC V4.0. Previous literature has suggested inconsistency between LS and VS in up to 25% of studies, yet it remains unclear whether routine use of VS offers any benefit to LS alone in diagnosing manometric abnormalities according to the CC V4.0.

**Aims:**

To determine if the routine use of VS improves the diagnostic yield in HR-EMS

**Methods:**

A retrospective analysis of all HR-EMS studies performed between December 2020 and July 2021 at Kingston Health Sciences Centre was completed. Demographic information including age, sex, indication for HR-EMS, surgical history, and chronic narcotic use was documented. Each study (consisting of 10 LS and 10 VS) was reviewed independently by a Gastroenterology Fellow and Neurogastroenterologist. A manometric diagnosis using CC V4.0 was made for both LS and VS. Descriptive statistics were performed.

**Results:**

A total of 101 HR-EMS studies were reviewed (33 male, 68 female, age range 26 to 90 years). The most common indication for HR-EMS was dysphagia (87/101) with 23/101 having 2 indications, 30/101 having 3 indications, and 43/101 patients having >3 indications. Prior upper GI tract surgery and chronic narcotic use was recorded in 9/101 and 8/101 patients, respectively. Two HR-EMS studies were excluded due to incomplete protocol. In total, 38.4% (38/99) had normal HR-EMS for both LS and VS. LS and VS that resulted in a CC V4.0 diagnosis were concordant in 37.4%(37/99) and discordant in 24.2% (24/99). Of the 24 discordant studies, 6 had a CC V4.0 diagnosis for LS (4 esophagogastric outflow obstruction (EGJOO), 1 ineffective esophageal motility (IEM), 1 diffuse esophageal spasm) and normal VS. Ten studies had normal LS and a CC V4.0 diagnosis for VS (9 IEM, 1 EGJOO). Eight had differing CC V4.0 diagnoses for LS and VS.

**Conclusions:**

LS and VS resulted in concordant diagnoses in the majority of cases. However, there were discordant results in approximately 25% of cases. In nearly half of these studies, the LS was within normal limits whereas VS yielded a diagnosis of an esophageal motility disorder which may be of clinical significance to the patient’s management. The addition of VS to HR-EMS protocol may increase diagnostic yield in symptomatic patients.

**Funding Agencies:**

None

